# Unnatural Amino Acid Crosslinking for Increased Spatiotemporal Resolution of Chromatin Dynamics

**DOI:** 10.3390/ijms241612879

**Published:** 2023-08-17

**Authors:** Pamela Moleri, Bryan J. Wilkins

**Affiliations:** Department of Chemistry and Biochemistry, Manhattan College, 4513 Manhattan College Parkway, Riverdale, NY 10471, USA

**Keywords:** unnatural amino acids, protein–protein crosslinking, posttranslational modifications, chromatin remodelers

## Abstract

The utilization of an expanded genetic code and in vivo unnatural amino acid crosslinking has grown significantly in the past decade, proving to be a reliable system for the examination of protein–protein interactions. Perhaps the most utilized amino acid crosslinker, *p*-benzoyl-(l)-phenylalanine (pBPA), has delivered a vast compendium of structural and mechanistic data, placing it firmly in the upper echelons of protein analytical techniques. pBPA contains a benzophenone group that is activated with low energy radiation (~365 nm), initiating a diradical state that can lead to hydrogen abstraction and radical recombination in the form of a covalent bond to a neighboring protein. Importantly, the expanded genetic code system provides for site-specific encoding of the crosslinker, yielding spatial control for protein surface mapping capabilities. Paired with UV-activation, this process offers a practical means for spatiotemporal understanding of protein–protein dynamics in the living cell. The chromatin field has benefitted particularly well from this technique, providing detailed mapping and mechanistic insight for numerous chromatin-related pathways. We provide here a brief history of unnatural amino acid crosslinking in chromatin studies and outlooks into future applications of the system for increased spatiotemporal resolution in chromatin related research.

In eukaryotic cells, DNA compaction is facilitated through the establishment of chromatin fibers, which are assembled through complexes of DNA, histones, and nonhistone proteins. The basic repeating unit of chromatin is the nucleosome, composed of DNA wrapped around a histone octameric core that induces negative supercoiling and the first level of DNA compaction in the nucleus. Each octameric core is composed of a single histone H3/H4 tetramer flanked by two histone H2A/H2B dimers, making contact with DNA through approximately 1.7 left-handed solenoidal twists [1,2]. DNA compaction and the nucleosomal unit have evolved to exist in an intriguing dichotomic state. On one hand, DNA must be easily accessible to enzymes that require access to genetic material, but, on the other hand, it must reside in a state of compaction to properly store DNA in the confines of the nucleus. Nucleosomal DNA breathes, allowing for fluctuating states between open and active, or closed and silent, chromatin. Changes in chromatin architecture and DNA access are coordinated through mechanisms that rely directly on the modified chemical state of histone proteins that ultimately regulate DNA spooling and unspooling mechanisms.

The histone family of proteins are well-known for their capacity to harbor posttranslational modifications (PTMs) that aid in critical chromatin mechanisms required for proper genome stability and function [3,4]. Local changes in chemical moieties on amino acid side chains induce distinct signals that lead to alterations in chromatin architectural maintenance. With the advancement of protein analytical techniques, in particular mass spectral analysis, the identification and characterization of histone PTMs has become quite extensive. Some of the most thoroughly characterized histone PTMs (e.g., acetylation, methylation, and phosphorylation) have been labeled as part of a “classical” subset of PTMs due to a multitude of recent novel modifications having been identified, providing a dizzying array of chemical combinations leading to unique histone regulatory pathways that have yet to be fully realized. For a more thorough overview of PTM variants and function, both classical and emerging, we refer you to the following reviews [5,6,7,8,9]. 

Chromatin dynamics are regulated through a cycle of PTM installments (requiring enzymatic “writers”), sequestering of protein contacts to the PTM for specific functional output (requiring protein “readers”), and PTM removal (requiring enzymatic “erasers”). The PTM cycle provides an effective system for protein interfacing at the surface of the nucleosome, where altering combinations of PTMs across each histone (varying at distinct chromosomal loci) creates a complex signaling pathway unique to each PTM pattern. While the mechanistic actions of the writers and erasers are imperative to our comprehensive understanding of chromatin behavior, it is the reader that becomes of particular interest because it is the functional device for output of the signaling pathway. These non-histone proteins bind, and/or maintain, epigenetic information that is stored and communicated through the histone modifications. There are still many unanswered questions regarding PTM dynamics and their regulation of chromatin fiber organization with a need for more details outlining how histone PTMs recruit non-histone proteins to the nucleosome. Additionally, the modifications often act in unison with other histone modifications, which together are proposed to establish a “histone code” for epigenetic memory [3,6]. It is the structural and functional relationship between histone PTMs, their modifiers, and readers, that is of fundamental importance to chromatin functional integrity. Accordingly, to truly appreciate how cellular regulation of chromatin is connected to PTM cycles, it is imperative to examine how the dynamic properties of histones regulate chromatin processes with spatiotemporal insight.

A fundamental class of nucleosomal reader proteins, the super family of chromatin remodelers, contributes an essential role in maintaining defined chromosomal architecture. Importantly, each class of remodeler is functionally influenced by defined PTM states across each histone. Remodelers possess histone binding domains that are localized to specialized chromatin regions corresponding to the histone modifications they recognize. ATP hydrolysis is then required to disrupt nucleosomal DNA contacts to slide, or displace, nucleosomes. ATP-dependent chromatin remodelers have five subfamilies, consisting of the SWI/SNF, ISWI, SWR1, CHD, and the INO80 remodeler complexes. They are classified by structural motif differences in their helicase, histone, and DNA binding domain [10] and regulate cellular processes such as transcription, replication, and differentiation [11]. Moreover, it is increasingly clear that chromatin remodeling complexes partake in essential roles in the DNA damage response pathways [12,13,14,15]. It is abundantly clear that remodelers shoulder a particularly important utility role for nucleosomal maintenance, and resolving their association with histone PTM patterns is paramount. 

The mechanistic details of the chromatin remodeler family are of particular interest because complexes must modify the chromatin landscape through opposing processes, establishing an elaborate interface with the chromatin fiber that is dependent on distinctive arrangements of PTMs (see the following reviews for an extensive overview of remodeler dynamics [10,16,17,18,19,20]). Remodeler complexes are large, multiunit protein structures that necessitate elaborate protein–protein stabilizing interactions. This makes it a difficult task to completely resolve their overall structures and molecular actions. Most mechanistic studies of chromatin remodelers, and chromatin in general, have been performed in solution studies. These works have detailed contacts that remodelers make to the nucleosome, but their relevance in vivo has been indirect [21,22]. Employing an in vitro chemical crosslinker to define protein–protein contacts is a powerful structural approach, but biological relevance is limited by the absence of a genuine nuclear environment.

While remodeler functional studies have been an emphasis of chromatin biology for decades, modern advances in structural studies have brought this class of proteins back into the spotlight. Recent reports have elegantly detailed high-resolution analyses of chromatin remodelers complexed with the nucleosome [23,24,25,26,27]. It is important to highlight that, even though these structures have provided the scientific community with stunning detail, many of the auxiliary subunit contacts to the nucleosome remain unresolved. Interestingly, part of the incomplete picture is a lack of resolution at the interface between remodeler subunit histone binding domains and the nucleosome. Furthermore, there is a need for techniques that provide greater biological relevance, in vivo, for these sizeable nucleosome complexed structures. Analyzing mechanistic details of not only remodelers but all nucleosome–protein contacts in real time in the living cell is a challenging task. Methods that address these types of exploration are important in order to clarify missing connections in our understanding of structural interfacing along the chromatin fiber. 

One way to approach some of the open questions in chromatin studies is through evaluation of protein–protein contacts and their dynamics under true physiological conditions. Examining the chromatin fiber in its nuclear environment allows us to gauge its native architectural structure and function. There remains an incomplete assortment of data regarding the intricate molecular interactions that are at play in, and around, the nucleosome, particularly in the context of the living nucleus. To this point, we provide a brief insight to chromatin analytical approaches using genetically installed unnatural amino acids (unAA) and a prospective strategy for broader application of this technique. 

An expanded genetic code allows for the expression of full-length protein harboring site-specific incorporation of unAAs that can act as unique chemical probes [28,29,30]. The method employs endogenous translational mechanisms to suppress an amber stop codon, utilizing an orthogonal pair of evolved aminoacyl-tRNA synthetase (aaRS) and suppressor tRNA. The aaRS/tRNA system creates a mechanism for unAA delivery to the ribosome and addition to a growing peptide chain. Using plasmid-borne expression vectors, containing the evolved aaRS/tRNA pair, live cells site-specifically install unAAs in response to the stop codon. This approach has far-reaching applications for protein studies; however, the chromatin field, specifically, became an important beneficiary of this method with the development of genetic encoding of PTMs that naturally occur on histones [31,32,33]. These advances allowed for the expression of full-length histones harboring distinct PTMs without the need for multistep peptide synthesis and chemical ligations. Not only are unAA PTMs of importance but also any probe that harbors chemical activity suitable for exploring protein–protein interfaces and their dynamic on/off states. 

Since the establishment of the expanded genetic code, now spanning two decades, the library of unAAs has grown quite extensively. Of interest to our work is the unAA *p*-benzoyl-(_L_)-phenylalanine (pBPA). This amino acid has proven to be a useful tool for studying chromatin dynamics in the living cell by taking advantage of its benzophenone side chain, which forms a diradical under UV exposure (~365 nm). The radical state allows for hydrogen abstraction and recombination with neighboring proteins that are within a distance of approximately 0.4 nm [29,34,35]. Significantly, for in vivo studies, pBPA has a low energy activation requirement that is not damaging to the cell, the crosslink is covalent allowing protein complexes to be readily isolated under various conditions, and control of the crosslinking event with UV light gives temporal directive to the researcher. When site-specific encoding and cell cycle synchronization is also considered, this crosslinking probe provides a formidable resource for detailed spatiotemporal insights to chromatin dynamics. 

The first detailed report of pBPA as an in vivo quantitative probe for histone studies investigated the dynamics of nucleosome–nucleosome interactions in *Saccharomyces cerevisiae* (Figure 1 provides a schematic overview of this process) [36]. The histone H2A acidic patch was scanned with pBPA and used to identify a protein–protein interaction with histone H4, which was previously characterized as being associated with condensed chromatin in vitro, but the biological relevance of the contact in vivo had not yet been addressed [1,37,38]. Sequentially, with the aid of synchronous cell populations, the H2A-H4 crosslink efficiency was revealed to reach its peak during mitosis. For the first time, the histone H2A-H4 interaction was directly correlated with stages of the cell cycle known to contain compact chromatin structures giving biological significance to the internucleosomal contacts. Quantitative crosslinking efficiency of this contact became an effective marker of compaction, revealing a temporal correlation of H4 K16 deacetylation and H3 S10 phosphorylation. Accumulation of H3 S10 phosphorylation during entry into mitosis was concurrent with the loss of H4 K16 acetylation and the accumulation of the histone H2A-H4 crosslink. 

These works led to a model of chromatin hypercondensation that was regulated by a cascade of PTMs, upon cellular commitment to mitotic entry. The model (Figure 2) proposed that histone H3 S10 phosphorylation worked as a sequestering signal for the deacetylase Hst2 (recently found to be mediated by the 14-3-3 protein, Bmh1, in yeast [39]), which then removed the acetylation mark on histone H4 at position lysine 16. The phosphorylation prompted the deacetylation of H4 K16, leading to an electrostatically charged histone H4 tail that was activated for binding to the H2A acidic patch. The model was the first establishment of histone H2A-H4 compaction dynamics in physiological conditions. The application of pBPA-histones provided a mechanism for chromatin condensation that had only been suggested in previous decades.

Since the application of this synthetic biology approach for chromatin investigations, histone crosslinking has been put into practice for a variety of studies. The H2A-H4 mitotic marker described above was further exploited, in combination with a microscopic assay for measuring distances in chromatin loci, to better understand the role of condensin proteins in the compaction process [40]. Assays revealed that H2A-H4 crosslinking efficiency remained unchanged in the presence of a heat sensitive deactivation of condensin, as the cells entered mitosis. This demonstrated that condensin was not required for short-range compaction of chromatin in mitosis. In another report that benefitted from the crosslinking system, pBPA was installed on heterochromatin protein 1 (HP1) to examine interfiber chromatin association and clustering [41]. This study helped delineate HP1 dimerization architecture when contacting the nucleosome, exposing a regulation of these interactions that was established by histone H3 lysine 9 trimethylation. In an alternative study, Hoffmann et al. employed pBPA to aid in establishing in vivo relevance for mechanistic actions of the yeast histone chaperone FACT (facilitates chromatin transcription), responsible for the removal of H2A-H2B dimers ahead of the transcription fork [42,43]. Utilizing pBPA scanning across two subunits of the FACT complex, Spt16 and Pob3, revealed that the C-terminal acidic tail of Pob3 crosslinked to H2A and H2B. Furthermore, data suggested that these interactions were negatively regulated by importin binding to a nuclear localization sequence that also resided on the C-terminus of the protein. In a more recent application of the crosslinking approach, Jain et al. used pBPA scanning to characterize the binding interface and mechanistic action of the yeast RSC remodeler complex ATPase subunit, Sth1, with the nucleosome (Figure 3) [44,45]. In this study, pBPA proved to be an effective spatiotemporal probe that allowed for detailed insights to Sth1 binding to histones. Ultimately, the work provided further explanation for the role of PTMs, histone H3 lysine 16 acetylation and H2B SUMOylation, during the sequestering and activation of the RSC complex. Data from this study provided in vivo clarification that Sth1 binding to the nucleosome is constitutive, challenging previous reports that assign the role of histone H3 acetylation as a sequestering signal for the RSC complex to the nucleosome. While deacetylated histone H3 lysine 16 does disrupt the binding of Sth1 to the terminal ends of the H3 tail, it does not hinder the binding of the protein to the H3 regions nearer the globular region or to the H2A acidic patch. Additionally, the study defined a translocation mechanism that is in line with the “sliding-mediated nucleosomal disassembly” model, where the *n* + 1 nucleosome is preferentially ejected, rather than the nucleosome to which the RSC complex is physically bound [46,47]. 

In total, these findings each highlight the versatility of the pBPA crosslinking system as a means for studying chromatin dynamics, both in vitro and in vivo. Importantly, the expanded genetic code system is applicable to any protein of interest allowing for chromatin exploration, not only in terms of histones, the aforementioned studies of HP1 and Pob1 being perfect examples. Importantly, the efficiency of crosslinked proteins can be readily quantified and used as a marker during mutational studies, particularly in terms of the influences of PTMs on the crosslinking event. Chromatin studies have unquestionably benefitted from the application of in vivo crosslinking utilizing unAAs, where it may also provide perspective advancements in other areas of chromatin-specific research. 

The application of pBPA for in vivo characterization of chromatin dynamics outlined above have been performed in the model organism *Saccharomyces cerevisiae*. This is for several reasons: (1) yeast genetics are easily and efficiently manipulated, (2) yeast is a eukaryotic system that allows for studies on histone proteins, (3) many cellular networking and signaling pathways are highly conserved from yeast to humans, and (4) genetic code expansion has been shown to be a powerful method for studying chromatin dynamics in the living yeast cell [36,48]. While trapping histone–protein interactions in yeast is well established at this point, the characterization of the histone binding partner requires a means of identifying the interaction following activation of the crosslinking event. There are two ways that this can be accomplished. The first is a candidate approach, where a known chromatin-related protein is tagged and subsequently assayed for the correct electrophoretic shift in a Western blot analysis. While this approach helped identify the H2A-H4 interactions during the establishments of these techniques, it is a biased approach that assumes the sized shift being queried matches that of a reasonable partnered interaction. It is the most feasible method for analysis of specific interaction targets; however, there are a myriad of known protein actors that bind along the same peptide stretches of the nucleosome, dependent upon the modification signal that is present for a specific pathway. Examination of whole cell lysates, following pBPA crosslinking, reveals a ladder of proteins that are UV-dependent [36,43]. Identification of each of the crosslinks in a particular sample using a candidate approach is unrealistic and impractical. 

Mass spectrometry (MS) provides an intriguing tactic for identifying the total histone interacting partners from a single crosslinking position. Crosslinked proteins are “locked” by a covalent bond and the protein–protein complexes can be readily isolated using an immunoprecipitation approach (i.e., HA-tagged histones in the reports mentioned here). The products, following analysis by SDS-PAGE, can be treated for tryptic digestion and then subjected to MS analysis. This approach allows histone crosslinked proteins to be assigned by identifying peptide sequences in the digested samples. Using this technique, whole cell lysate preparations of more than ten sites along both histone H3 and H2A has revealed the identity of numerous known chromatin-associated proteins, as well as potentially novel contacts (unpublished data), providing preliminary data supporting the viability of this technique. This scheme limits the need for a candidate approach allocation of protein partner assignments. Surveying all crosslinks within a single sample reveals how multiple proteins interact at a defined nucleosomal residue along a chromosomal fiber that is under distinctively different regulation at multiple locally specialized chromatin structures and becomes a notable tool for the identification of histone–protein interactions. A recent review of MS approaches to chromatin studies elegantly details various advances in methodologies specific to histone and PTM dynamics [49], yet the use of pBPA in these approaches is absent. Utilizing pBPA for protein–protein studies, across numerous species, has been well documented [29,36,50,51,52,53,54], with a myriad of protein–protein interaction studies explicitly aimed at chromatin dynamics [55]. Surprisingly, the use of pBPA for MS analysis and characterization of chromatin dynamics has yet to be fully explored and has the potential to be a powerful pairing for MS structural analyses. In addition, an isotopically labelled pBPA molecule would serve as an intriguing companion to this approach by providing isotopic foot printing for confident identification of parental peptide masses [56]. Peptide sequencing data provides a higher resolution mapping of the interactome at the nucleosomal surface and offers an unbiased proteomic approach that can detect several binding partners from one sample, as well as identify new chromosomal binding factors.

In another facet of commissioning the unAA system, it is imperative to point out that the incorporation of pBPA into the chromatin landscape cannot control which nucleosomes acquire the crosslinking agent. The method does not require the removal of genomic copies of histones, and therefore the chromosome contains both mutant and wild-type histone proteins in the global chromatin landscape. The researcher retains spatial probe control for the positioning within the protein but loses this control in terms of spatial nucleosomal distribution. Because of this, it is difficult to assess the chromosomal positioning of an identified nucleosomal contact. Higher resolution spatiotemporal models of protein–nucleosome binding maps will rely on knowing the local chromatin architecture and processes involved. While it may be plausible that complexed crosslinking interacts the same regardless of the local environment, these issues must be addressed. 

Chromatin immunoprecipitation (ChIP) is the gold standard for studying protein association with DNA and characterization of protein distribution across the genome [57,58,59]. While this technique has been proven to be a powerful ally for the delineation of proteins associated with specific DNA sequences, notably the signature of a multitude of promoter elements and their correlation to histone PTMs, the limitations of ChIP become apparent when trying to interpret mechanistic details at the site of interest. The chemical crosslinking used for ChIP is nonspecific, and higher resolution is granted to strong DNA binding partners. Many proteins act at the nucleosome rather transiently, relying mainly on protein–histone contacts, or other bridging mechanisms, rather than protein–DNA contacts, reducing the efficiency in which ChIP can accurately report on certain non-histone nucleosomal interactions. Chromatin remodelers, for example, are notoriously difficult to analyze via ChiP protocols due to their dynamic binding, involving cycles of structure fluctuation during translocation events. Importantly, many of the strongest contacts that remodelers make with the nucleosome are through histone contacts rather than DNA. We envision a combination of the pBPA photo-crosslinking system and ChIP as a means to increase functional remodeler mechanistic insights. 

Recent advances in our own work have focused on isolating DNA fragments that are associated with the immunoprecipitation of the crosslinker histone, exploiting its HA epitope. Following standard expression of pBPA-histones permits for the random distribution of the pBPA probe across the native chromosomal landscape. Considering that the crosslinks of interest may be expressed from a genetic background that provide epitope-tagged versions of target proteins (i.e., subunits of chromatin remodelers or any other transient DNA interacting protein), an initial photocrosslinking event can be stimulated to ensnare a histone–protein interaction. Following photocrosslinking, standard ChIP chemical crosslinking and preparations can be applied. In this proposed system, DNA is stably crosslinked to the tagged histone, which is crosslinked to the target protein via a stable bridging contact. An initial HA-precipitation of the histone-associated DNA fragments are enriched for specific crosslinks by immunoprecipitation with the crosslinked target’s epitope antibody (Figure 4). The enrichment of the epitope-associated DNA sequences will clarify the positions of the crosslinked protein along the fiber, without having to be specifically bound to DNA itself. This approach is expected to be a powerful tool to assess nucleosome occupancy of proteins that interact at the nucleosome more transiently and are difficult to pull down in normal coprecipitation reactions. While there are several other ChIP-based techniques that have been successfully used to study remodeler protein chromosomal occupancy [60,61,62,63,64,65], we believe this approach will simplify the process, as well as provide layers of spatial detail (both histone crosslinking and nucleosomal positioning) in a single assay. 

Understanding the global incorporation of the pBPA-histones alone would be of great importance, to ensure there is no positioning bias for the distribution of pBPA across the genome. To this end, preliminary ChIP and qPCR results from our lab suggest that pBPA is indeed distributed across random chromosomal loci (unpublished). Collectively, when ChIP is paired with spatiotemporal control of pBPA crosslinking, a truly comprehensive model of the protein–nucleosome complexes can be resolved, under physiological conditions.

An interesting direction for the evolution of this technique is a deeper examination of DNA damage pathways and understanding how chromatin remodelers contribute. DNA damage signaling promotes broad changes in histone modifications and the recruitment of nucleosomal remodeling complexes. How histone modifications control remodeler interactions at the nucleosomal interface during the response pathways is rather vague. An example is the yeast RSC remodeler as a regulatory component of the base excision repair pathway (BER) [15]. Using a technique to control the conditional knockdown of the Sth1 subunit, researchers revealed an increased sensitivity to DNA damaging agents and a reduced rate of repair. The results are the first in vivo implication that RSC plays an important role in the repair pathway, where previous solution studies demonstrated the significance of RSC on a reconstituted BER nucleosomal template [66]. The results together correlate the remodeler activity with BER, but the molecular regulation and mechanism of the complex at the nucleosome is absent. When BER is initiated, how is RSC signaled to the chromosome? What histone modifications are installed or removed upstream of the localization of the complex? These molecular details are missing components of the functional dynamics of such pathways. 

Interestingly, RSC has also been shown to be a factor in the homologous recombination and non-homologous end joining repair (NHEJ) pathways of DNA double-strand breaks [67,68]. This implies that remodeling complexes are functioning across a multitude of pathways and therefore must be precisely regulated for each. Moreover, several chromatin remodeler complexes from different families have been shown to play a role in the same damage pathways. It is unclear how many distinctive remodeling complexes act within a specific pathway, and which remodeler actions are redundant versus having explicit functionality. It is also ambiguous as to which remodelers act at which steps in each of the response pathways and whether they function up or downstream of the signal/repair process. Using pBPA approaches to study these spatiotemporal dynamics would be of great consequence for future DNA damage-related disease studies and would help shape future therapeutic outlooks. 

While the pBPA genetic code expansion system provides a quantitative approach to in vivo crosslinking, it does have limitations that are worth mentioning. Proper unAA suppression of the amber stop codon, in the living cell, is in competition with the ribosomal release factor. Because of this conflict, full-length protein is produced at a fraction of that observed for control levels (roughly ten percent of total protein for in vivo histone experiments [36]). Additionally, the benzophenone chemistry requires appropriate distancing and geometry for proper radical recombination reactions. Taken together, the pBPA system suffers from rather subdued crosslinking yields, and low throughput, as each pBPA position scan requires its own individual cellular assay. Additionally, pBPA incorporation may interrupt cellular physiology, requiring fitness testing for each individual process being studied. Nevertheless, even with its limitations, the system is readily optimizable for signal output and reproducible quantitative assessments, providing a unique approach to studying protein dynamics in their natural environments. 

In conclusion, the study of histone–protein interactions using an expanded genetic code has provided a plethora of new insights to chromatin biology over the past decade. This synthetic biology tool not only provides spatial details for protein–protein contacts but also adds a level of temporal control due to the UV activation of the probe. When this is paired with synchronous cell populations, studying distinct crosslinking events allows for the quantitative analysis of the interaction over the course of the cell cycle. Additionally, the crosslink itself serves as a marker, where crosslinking efficiency changes are a direct reflection of a system modifier, such as deletion and mutational analysis. One of the most important aspects of this work is that it provides a platform to study protein sequestering, and protein–protein dynamics, in relationship to PTMs, particularly in respect to chromatin remodelers. These large complexes are difficult to study in the living cell, and our system provides an elegant means to address remodeler dynamics with biological significance. Lastly, with an influx of new PTMs being introduced each year, this approach provides a promising means of exploration in this area to keep pace with advances in structural analytic techniques such as MS and cryo-EM. 

## Figures and Tables

**Figure 1:**
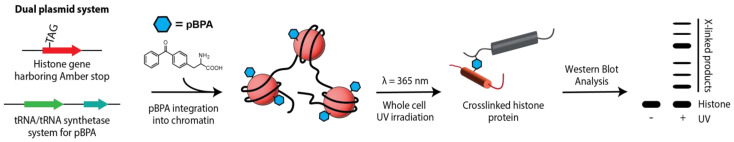
pBPA-histone expression and crosslinking scheme. A dual plasmid system is utilized for the expression of a histone gene harboring an amber stop codon at the desired position of interest and the genetic code expansion tRNA synthetase/tRNA system for the incorporation of pBPA. pBPA-histones are allowed to integrate into the native chromatin landscape, and then whole cells are exposed to UV radiation in order to activate the crosslinker. Captured protein–protein interactions are then analyzed via Western blotting. Whole cell lysate analysis yields a ladder of UV-dependent proteins that can be characterized via mass spectrometry. Alternatively, an epitope tagged target protein can be employed to query specific histone–protein contacts through immunoprecipitation of the target and decorating for the coprecipitated histone.

**Figure 2:**
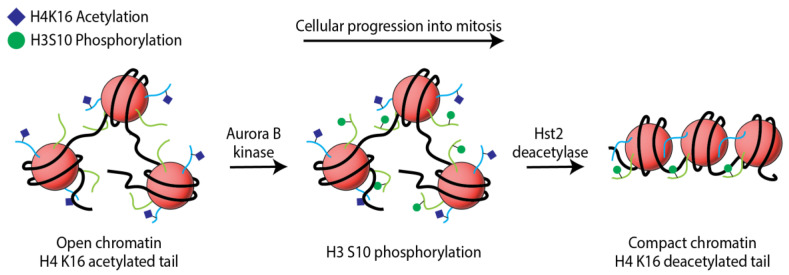
Overview for model of PTM cascade involved in yeast chromatin condensation during mitotic entry. H4 K16 acetylation is at its maxima during interphase when chromatin architecture is open. The acetylation mark neutralizes the basic charge of the histone tail disrupting electrostatic attractions with the H2A acidic patch of neighboring nucleosomes. As cells progress into the G2/mitotic phases, histone H3 S10 is phosphorylated (H3 S10ph), reaching its maxima in mitosis. H3 S10ph signals binding of Hst2 deacetylase, which in turn removes the acetylation mark on H4. The H4 tail makes contact with the H2A acidic patch causing compaction of the nucleosomal units.

**Figure 3:**
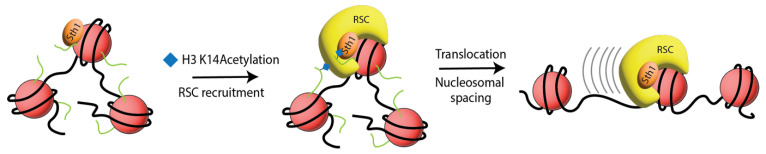
Model for Sth1 binding, RSC recruitment, and translocation. Sth1 is constitutively bound to nucleosomes through contacts on the H2A acidic patch and the histone H3 tail at position S22. Acetylation signals Sth1 binding to the terminal ends of histone H3, accumulation of the RSC complex at the nucleosome, and then translocation of neighboring nucleosomes to establish nucleosome-free regions at the promoter.

**Figure 4:**
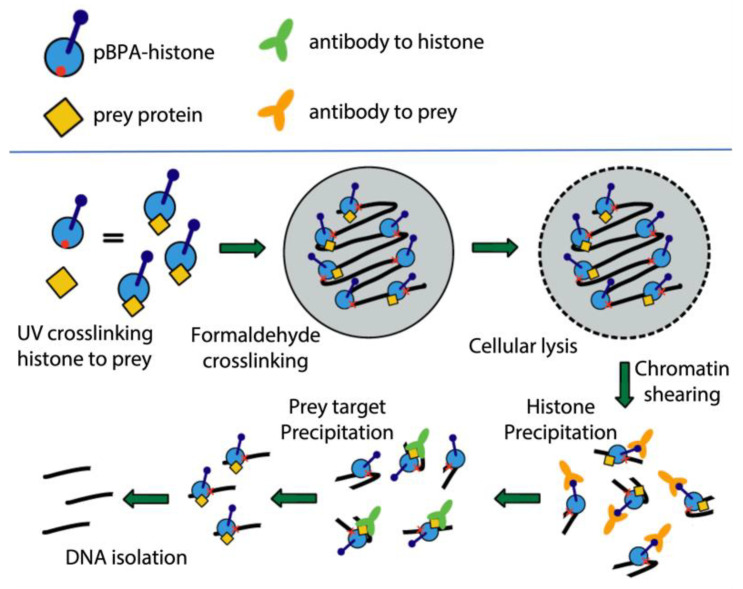
Schematic overview of pBPA-histone expressions paired with ChIP applications for higher resolution of transient chromatin binding proteins. This double crosslinking, double IP, approach clarifies DNA associated with proteins that make stable contacts with histone proteins, rather than DNA.

## Data Availability

No new data were created or analyzed in this study. Data Sharing is not applicable to this article.
